# A Novel Role of Annexin A2 in Human Type I Collagen Gene Expression

**DOI:** 10.1002/jcb.24989

**Published:** 2015-01-20

**Authors:** Georgia Schäfer, Jessica K. Hitchcock, Tamlyn M. Shaw, Arieh A. Katz, M. Iqbal Parker

**Affiliations:** ^1^ Division of Medical BiochemistryFaculty of Health SciencesUniversity of Cape TownCape TownSouth Africa; ^2^ Institute of Infectious Disease and Molecular Medicine (IDM)University of Cape TownCape TownSouth Africa; ^3^ International Centre for Genetic Engineering and Biotechnology (ICGEB)Cape TownSouth Africa

**Keywords:** annexin a2, collagen modulating element, extracellular matrix, type i collagen

## Abstract

The fibrillar collagen scaffold of the extracellular matrix provides a structural framework for cells in tissues and regulates intercellular communication; its disregulation has been associated with tumour development and progression. Previous work has shown that expression of type I collagen, the most abundant mammalian extracellular matrix protein, is decreased in chemically or virally transformed cells. This negative regulation could be mapped to a proximal COL1A2 promoter element spanning a CME (Collagen Modulating Element) site in SV40‐transformed human fibroblasts (SV‐WI38) that binds an unknown repressing protein. By magnetic bead pull‐down, we observed a multi‐protein complex bound to the CME with preference for single‐stranded over conventional double‐stranded DNA. MALDI‐TOF mass spectrometry of the CME‐binding protein complex revealed involvement of nuclear annexin A2 (AnxA2) which was increased in SV40‐transformed cells. Further EMSA analysis demonstrated that AnxA2 did not directly bind to the DNA but stabilised the complex and led to an increase in protein binding to the CME in SV‐WI38 but not untransformed WI38 cells. Knockdown of AnxA2 by siRNA increased type I collagen production in both WI38 and SV‐WI38 cells; however, these effects were not mediated at the transcriptional level. Rather, our data indicate a novel functional role of AnxA2 in the negative post‐transcriptional regulation of type I collagen synthesis in human fibroblasts. In SV40‐transformed cells, AnxA2 is accumulated at the proximal COL1A2 promoter region, suggesting close association with the transcriptional machinery that possibly facilitates binding to the emerging mRNA, eventually contributing to overall repression of type I collagen protein synthesis. J. Cell. Biochem. 116: 408–417, 2015. © 2014 The Authors. *Journal of Cellular Biochemistry* published by Wiley Periodicals, Inc.

**Abbreviations**
AnxA2: annexin A2
CME: collagen modulating element
Co‐IP: coimmunoprecipitation
COL1A2: α2(1) collagen chain
EMSA: electrophoretic mobility shift assay
SV40 T Ag: SV40 large T antigen
SV‐WI38: SV40 transformed WI38 cells
TCEP: tris (2‐carboxylethyl) phosphine hydrochloride

Type I collagen is the most abundant mammalian extracellular matrix protein and consists of a heterotrimer of two α1(1) and one α2(1) chains. Its expression is regulated by both transcriptional and translational events, as this is a prerequisite for the normal functioning of the connective tissue [Rossert et al., 2000]. However, in pathological situations such as cancer, type I collagen levels are altered, and the degradation of the extracellular matrix is believed to be important for tumour invasion and metastasis [Fenhalls et al., 1999; Jinka et al., 2012; van Rooyen et al., 2013]. Indeed, it has long been known that cellular transformation leads to a marked reduction in type I collagen levels with α2(1) chains being preferentially lost, leading to low amounts of an unstable homotrimer of the α1(1) chain [Parker et al., 1989]. Previous work from our laboratory has led to the identification of two elements in the proximal promoter of the COL1A2 gene, which encodes the α2(1) collagen chain, that are essential for its basal activity [Parker et al., 1989, 1992; Collins et al., 1997, 1998]: the inverted CCAAT box (CCAAT‐Binding Element, or G/CBE) and the adjacent Collagen Modulating Element (CME). While the CBE was found to bind the heterotrimeric CCAAT‐Binding Factor (CBF) that activates COL1A2 gene expression, the CME binds an as yet uncharacterised DNA‐binding protein [Collins et al., 1997]. It is hypothesised that the CME in the COL1A2 promoter has a context‐ and species‐specific regulatory function as this element is only present in the human promoter [Collins et al., 1997; Leaner et al., 2005]. This is supported by the observation that in the COL1A2 expressing human lung fibroblast cell line WI38, a transcriptional activator binds to the CME and probably functions cooperatively with the adjacent CBE binding factors in the regulation of the COL1A2 gene [Collins et al., 1997]. However, upon WI38 fibroblast transformation with the DNA tumour virus SV40 (termed SV‐WI38), COL1A2 gene expression is markedly decreased [Parker et al., 1989], and the CME was found to bind additional nuclear protein(s) with repressor functions that are responsible for the gene's downregulation in this cell line [Parker et al., 1992].

Efforts to identify the CME binding factor(s) in SV‐WI38 cells led to the characterisation of at least one protein with a molecular weight of 66 kDa that possibly constituted a heteromultimeric complex that required Zn^2+^ for binding [Collins et al., 1998]. In the present study, we identified Annexin A2 (AnxA2) as part of the CME binding complex that is involved in the regulation of type I collagen expression. AnxA2 is a multi‐functional protein that belongs to a large family of Ca^2^^+^‐dependent cytosolic phopsholipid‐ and membrane‐binding proteins. Apart from their proposed roles as membrane–membrane or membrane–cytoskeleton linkers, AnxA2 has been implicated in diverse cellular functions, including Ca^2^^+^‐dependent regulation of exocytosis, DNA synthesis and cell proliferation [Hitchcock et al., 2014]. Interestingly, AnxA2 was shown to have regulatory RNA‐binding capacities [Kwak et al., 2011], to exert DNA‐binding activities [Boyko et al., 1994], to contribute to post‐transcriptional regulation of the expression of specific genes [Vedeler et al., 2012], to mediate interactions with protein ligands and to promote nuclear entry of the transcription factor Signal Transducer and Activator of Transcription 6 (STAT6) [Das et al., 2010].

Here, we describe a novel functional role of AnxA2 in the post‐transcriptional regulation of human type I collagen expression contributing to its decrease upon malignant transformation.

## MATERIALS AND METHODS

### CELL CULTURE

WI38 human embryonic lung fibroblasts (ATCC) and SV‐WI38 (SV40 transformed WI38 cells, [de Haan et al., 1986]) were grown in DMEM (Invitrogen) supplemented with 10% heat‐inactivated fetal calf serum (Invitrogen), 100 U/ml penicillin and 100 µg/ml streptomycin in a humidified atmosphere (5% CO_2_) at 37 °C.

### RNA EXTRACTION AND QUANTITATIVE RT‐PCR

Total RNA was extracted from WI38 and SV‐WI38 cells with TRIzol reagent (Invitrogen), DNAse‐treated and reverse transcribed using the ImProm‐II™ Reverse Transcription System (Promega). cDNA generated from 1 µg of total RNA was used for quantitative PCR with the SensiMix SYBR No‐ROX Kit (Bioline) on a LightCycler®480II System (Roche). Products were amplified with primers for human COL1A2 [Buttner et al., 2004] and AnxA2 [Tabata et al., 2006], respectively, normalised to the reference genes β‐actin [Meerbach et al., 2001] and HSPC3 [Krainova et al., 2013], and analysed using the software qbase+ (biogazelle).

### WESTERN BLOT AND CO‐IMMUNOPRECIPITATION

Cytoplasmic and nuclear proteins were prepared from WI38 and SV‐WI38 cells using the NE‐PER® Nuclear and Cytoplasmic Extraction Reagents (Thermo Scientific) and analysed in SDS‐PAGE and Western blotting according to conventional protocols. Membranes were probed with the primary antibodies goat anti‐type I collagen (Southern Biotech), mouse anti‐AnxA2 (R&D Systems), mouse anti‐STAT6 (BD Biosciences) and rabbit anti‐GAPDH (Cell Signaling Technology, Inc.), respectively, followed by incubation with the appropriate horseradish peroxidase (HRP)‐conjugated secondary antibody. Protein levels were visualised by chemiluminscence using the LumiGlo® Reserve Substrate (KPL) and the VisionWorks LS Biospectrum™ 500 Imaging System (UVP). The same membrane was used for multiple protein detection by stripping with 0.1 M glycine‐HCl (pH 2.2) and reprobing with the appropriate antibody.

Co‐immunoprecipitation experiments were performed using the Pierce® Co‐Immunoprecipitation (Co‐IP) Kit (Thermo Scientific). Briefly, 300 μg of nuclear protein extracts prepared from SV‐WI38 cells in a total volume of 300 μl IP Lysis/Wash Buffer (“Input” fraction) were incubated with 5 μg of a mouse antibody against SV40 large T antigen (SV40 T Ag; Santa Cruz) with gentle mixing overnight at 4 °C, followed by precipitation with 50 μl Protein G Plus/Protein A Agarose (Oncogene) for a further 2 h at 4 °C. Unbound proteins were collected as “Flow‐through” fraction, while the agarose beads were washed five times with Lysis/Wash Buffer after which bound proteins were eluted in a total volume of 30 μl Elution Buffer (“Eluate”). Co‐IP experiments were analysed by Western Blotting using the antibodies mouse anti‐SV40 T Ag (Santa Cruz), mouse anti‐AnxA2 (R&D Systems) and rabbit anti‐p53 (Santa Cruz), respectively.

### ELECTROPHORETIC MOBILITY SHIFT ASSAY (EMSA)

**Figure 1:**
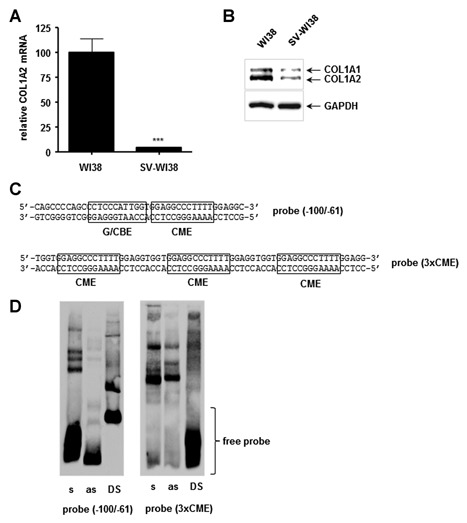
**Differential binding of nuclear proteins to COL1A2 promoter probes**. (A) Quantitative Light Cycler RT‐PCR showing downregulation of the human COL1A2 gene in SV‐WI38 compared to wildtype WI38 cells which was set as 100%. Experiments were performed in triplicates and normalised to β‐actin and HSPC3 expression, *P* < 0.001. (B) Western Blot confirming decreased type I collagen protein in SV‐WI38 cells; GAPDH served as loading control. (C) Probes used in EMSAs. The probe (−100/−61) represents the COL1A2 promoter region from −100 to −61 relative to the transcriptional start site that contains an inverted CCAAT Binding Element (CBE) and an adjacent Collagen Modulating Element (CME). The probe (3xCME) consists of the CME consensus sequence repeated three times. (D) EMSA showing the binding of SV‐WI38 nuclear proteins to either the (−100/−61) probe or the (3xCME) probe used either single‐stranded (s = sense or as = antisense), or double‐stranded (DS), respectively. Note that the position of the free probe differs between single‐stranded and double‐stranded oligonucleotides.

Electrophoretic mobility shift assay (EMSA) analysis was performed non‐radioactively using the Light Shift® Chemiluminescent EMSA Kit (Thermo Scientific). Briefly, nuclear extracts were prepared from WI38 and SV‐WI38 cells with the NE‐PER® Nuclear and Cytoplasmic Extraction Reagents (Thermo Scientific), while DNA‐probes were labelled with biotin‐11‐dUTP using the Biotin 3′ End DNA Labeling Kit (Thermo Scientific). The sequences of the probes are depicted in Figure 1C; they were used either single‐stranded or double‐stranded as indicated. 3 µg of nuclear protein extract and 200 fmol of the biotinylated probes were incubated at room temperature for 15 min in EMSA binding buffer containing 10 mM Tris (pH 7.5), 50 mM KCl, 1 mM DTT, 1 mM zinc acetate, 2.5% glycerol, 5 mM MgCl_2_ and 50 ng/µl poly‐dIdC in a total volume of 20 µl. Where indicated, 0.1–0.5 µg of recombinant human AnxA2 protein (US Biological) was added to the binding reaction. For supershift assays, 2 µg of anti‐AnxA2 antibody (R&D Systems) or anti‐STAT6 (S‐20) (Santa Cruz) which served as negative control, was incubated with the nuclear extracts for 15 min at room temperature before adding the probe. After incubation, DNA‐protein complexes were resolved in 10% native polyacrylamide gels, transferred to a positively charged nylon membrane (Hybond‐N + , Amersham Biosciences) and cross‐linked under UV light using a Spectrolinker XL 1000 machine (Spectronics Corporation) at 120 mJ/cm^2^ for 2 min. The biotin‐labelled DNA signals were visualised using the Light Shift® Chemiluminescent Nucleic Acid Detection Module (Thermo Scientific) with the VisionWorks LS Biospectrum™ 500 Imaging System (UVP).

### BIOTIN‐STREPTAVIDIN MAGNETIC BEAD PULL‐DOWN ASSAY

**Figure 2:**
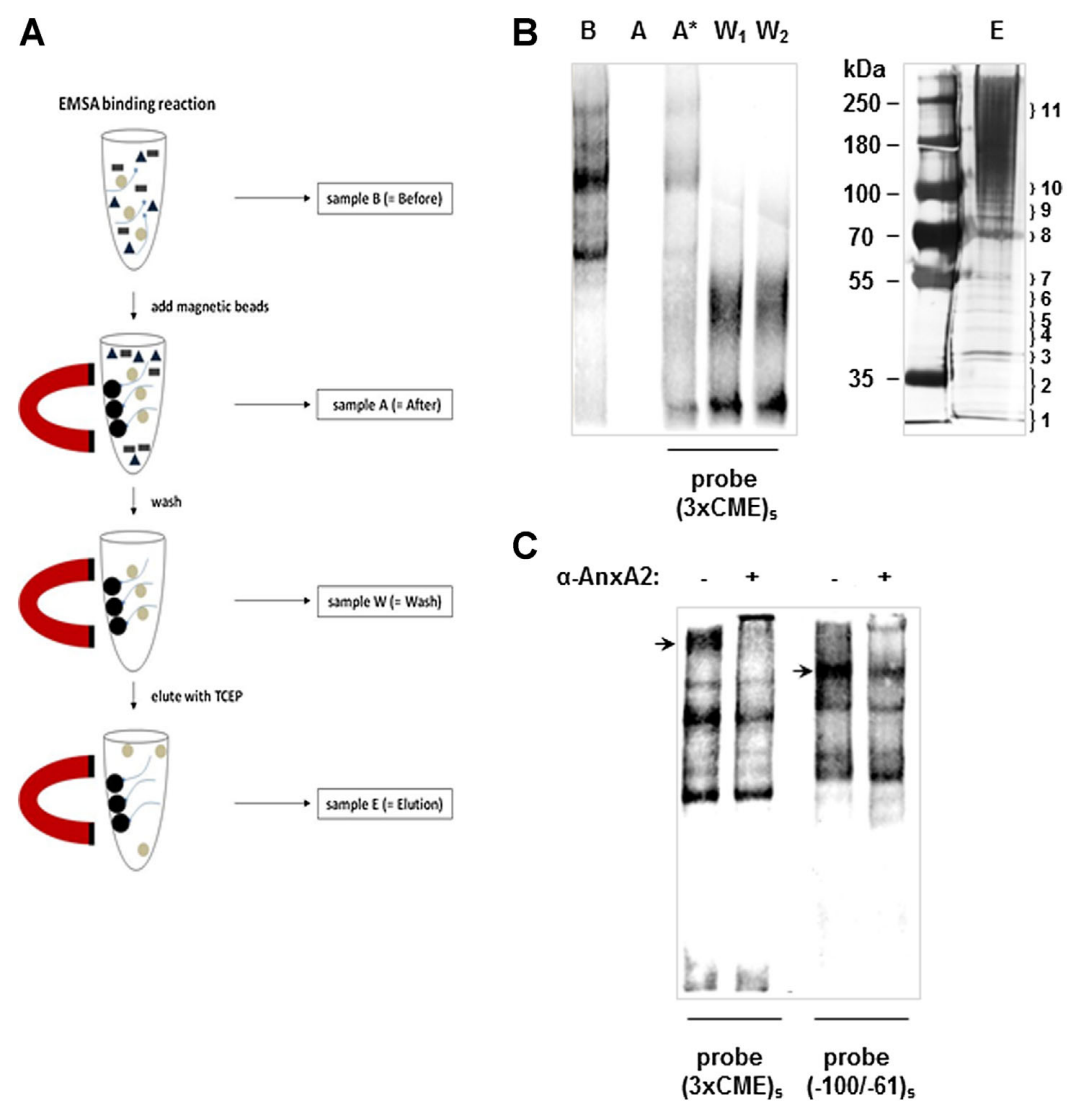
**Isolation of CME binding proteins with Magnetic Bead Pull‐Down.** The outline of the biotin‐streptavidin magnetic bead pull‐down assay is depicted in (A) with samples taken at various points for analysis in EMSA and SDS–PAGE as shown in (B). Briefly, SV‐WI38 nuclear proteins were incubated with the biotinylated single‐stranded (3xCME)_s_ probe under optimised EMSA binding conditions. A sample was taken (B = Before) for EMSA analysis. Streptavidin‐coated magnetic beads were then added to remove the biotinlyated probe with bound protein complexes from solution by magnetic separation; a sample (A = After) was taken for EMSA analysis. To account for loss of probe and allow direct comparison of binding reactions, an appropriate amount of biotinylated probe was added in the EMSA (A* = After, with added probe). Protein complexes bound to the probe were washed twice and recovered by magnetic separation while the remaining supernatant was analysed by EMSA (W_1_ and W_2_ = Wash 1 and 2). Protein complexes were eluted from the probe with 250 mM TCEP and separated by SDS–PAGE with subsequent silver staining (E = elution). Numbers on the right correspond to the gel fragments that were cut out from the gel and subjected to MALDI–TOF mass spectrometry analysis. (C) EMSA showing blocking of complex formation to the (3xCME)_s_ probe and the (−100/−61)_s_ probe, respectively, when SV‐WI38 nuclear proteins were incubated in the presence of an anti‐AnxA2 antibody. Major protein complexes interacting with the antibody are indicated with an arrow.

Nuclear extracts were prepared from SV‐WI38 cells as described above. 750 µg of protein was incubated with 50 pmol of the biotinylated 3xCME probe (sense strand) for 15 min at room temperature in EMSA binding buffer (see above) in a total volume of 5 ml. A 20 µl aliquot of the binding reaction was removed and the success confirmed by EMSA (see outline Fig. 2A). Following the incubation, 10 mg of streptavidin magnetic particles (Roche) were added to the reaction, incubated at room temperature for 30 min with gentle shaking and subsequently recovered with a magnet. To visualise the efficiency of the binding of the biotinylated probe (with bound proteins) to the magnetic beads, two 20 µl aliquots were removed for EMSA analysis [one aliquot did not receive any probe to confirm removal of the probe (i.e., binding to the beads), while the other aliquot was replenished with an appropriate amount of probe to confirm removal of DNA‐binding protein complexes]. The protein‐DNA‐streptavidin‐magnetic particles were washed twice with EMSA binding buffer, and 20 µl aliquots were removed for EMSA analysis (with an appropriate amount of probe added) to confirm protein binding to the magnetic beads during the washing steps. Bound proteins were eluted with 250 mM of the mass spectrometry compatible denaturing agent TCEP (Tris [2‐carboxylethyl] phosphine hydrochloride, Sigma) and further purified and concentrated using Amicon Ultra‐0.5 ml Centrifugal Filters 3 K devices (Millipore). Eluted proteins were then separated by SDS–PAGE using 10% acrylamide and visualised by silver staining with the Pierce® Silver Stain Kit (Thermo Scientific). To identify separated proteins, individual bands were cut out from the gel and further processed for analysis by MALDI–TOF mass spectrometry at the CPGR (Centre for Proteomic & Genomic Research, Cape Town, South Africa). Proteins were identified from the mass spectrometry data using Matrix Science database and searching the NCBI database.

### siRNA MEDIATED SILENCING OF GENE EXPRESSION

WI38 and SV‐WI38 cells were reverse transfected with Silencer® Select siRNA (Applied Biosystems) for AnxA2 using the TransFectin Lipid Reagent (Bio‐Rad). Briefly, 8 µl of a 20 µM siRNA stock was diluted in 100 µl serum‐free DMEM and added to 1.5 µl transfection reagent diluted in 100 µl serum‐free DMEM. The mixture was incubated at room temperature for 30 min, dispensed into a clean 3.5 cm culture dish and gently mixed with 1.8 ml of a prepared cell suspension of 2 × 10^5^ cells in DMEM (containing 10% fetal calf serum) to give a final concentration of 80 nM siRNA. Cells were incubated for 48 h at 37 °C under normal culture conditions and then processed for Western Blot or quantitative RT‐PCR analysis.

## RESULTS

### DIFFERENTIAL NUCLEAR PROTEIN BINDING TO THE CME AT THE PROXIMAL COL1A2 PROMOTER

While human embryonic lung WI38 fibroblasts produce normal levels of type I collagen, SV‐WI38 show a marked reduction in COL1A2 gene expression [Parker et al., 1989]. We confirmed this long‐known observation by quantitative RT‐PCR (Fig. 1A) and found that the COL1A2 gene was significantly downregulated to approximately 5% in SV‐WI38 cells compared to untransformed cells. This was also confirmed at the protein level by Western Blot analysis where type I collagen production was reduced in SV‐WI38 cells to approximately 20% (Fig. 1B).

The human COL1A2 gene is transcriptionally regulated via a proximal promoter element spanning the CBE and the CME [Parker et al., 1989, 1992; Collins et al., 1997, 1998]. Previous work indicated that the transcriptional downregulation of the COL1A2 gene in SV‐WI38 is due to an unknown repressor protein or protein complex that bound the CME located in the proximal COL1A2 promoter region between −100 and −61 relative to the transcriptional start site [Parker et al., 1992; Collins et al., 1998]. We therefore set out to optimise the binding conditions between SV‐WI38 nuclear extracts and a synthetic COL1A2 promoter element spanning the CME in order to isolate the binding protein(s). In addition to the actual −100 to −61 promoter region, we also generated a DNA probe containing three repeats of the CME [termed (3xCME), Fig. 1C] in order to increase protein binding and to overcome any steric hindrance when coupled to magnetic beads in subsequent magnetic bead pull‐down assays. Early work on COL1A2 suggested that the promoter region is capable of forming secondary hairpin structures that are possibly involved in transcriptional regulation [Collins et al., 1997; Dickson et al., 1985]. We therefore tested the effect of protein binding to both single‐stranded as well as double‐stranded DNA probes. As shown by EMSA in Figure 1D, both probes strongly bound SV‐WI38 nuclear proteins when in the single‐stranded sense form; the corresponding anti‐sense form did not bind nuclear proteins when the (−100/−61) probe was used, while the (3xCME) probe did bind but at a much lesser extent. Note that differences in the binding pattern are due to the different probe sizes and enhanced complex binding when the three elements are present. The binding to the double‐stranded probes was either less intense (for the [−100/−61] probe) or did not lead to distinct complex formation at all [for the (3xCME) probe] compared to the corresponding single‐stranded sense probes. As this observation strongly suggests that the CME binding protein(s) in SV‐WI38 cells prefer single‐stranded DNA over the conventional double‐stranded helix, we continued with the purification of the CME binding protein(s) using the single‐stranded probe (3xCME) in the sense orientation (referred to as [3xCME]_s_).

### ISOLATION OF CME BINDING PROTEIN COMPLEXES WITH (3x CME)_s_ LINKED TO MAGNETIC BEADS

In order to isolate the CME‐binding protein(s), we applied optimised EMSA conditions to allow nuclear protein binding to the biotinylated (3xCME)_s_ probe which was then immobilised to streptavidin‐coated magnetic beads (Fig. 2A). To assay the amount of binding proteins before magnetic separation and to visualise the efficiency of the magnetic bead coupling to the (3xCME)_s_ probe, reference aliquots for EMSA analysis were taken before and after the coupling (Fig. 2B). The sample taken before coupling to the magnetic beads shows normal DNA‐protein binding pattern (Fig. 2B, sample B), while no signal could be detected when the biotinylated probe with bound protein was removed by the streptavidin‐coated magnetic beads (Fig. 2B, sample A). To compensate for the loss of probe and to evaluate the specific removal of CME‐binding proteins by the magnetic beads, an appropriate amount of (3xCME)_s_ probe was added to sample A and visualised by EMSA (Fig. 2B, sample A*). To remove non‐specifically bound factors, the beads were washed twice and aliquots were taken to confirm that there was no loss of CME‐binding proteins (Fig. 2B, samples W_1_ and W_2_). Finally, to elute the bound proteins from the probe, we chose a condition that dissociates DNA‐protein complexes and used a high concentration (250 mM) of the mass spectrometry compatible denaturing agent TCEP. Eluted proteins were separated by SDS–PAGE (Fig. 2B, sample E) and a total of 11 gel fragments containing dominant protein bands were cut out after silver staining and analysed by MALDI–TOF mass spectrometry. The obtained mass spectra were compared against the Matrix Science Database (MSDB) and are listed in Table I. Since there were more than one excised band per gel fragment in several cases (Fig. 2B, lane E), some of the hits listed in Table I indicate different proteins per gel fragment. Among the molecules identified, we considered AnxA2 as an attractive candidate involved in COL1A2 regulation because of its known nuclear localisation [Das et al., 2010] and its biological function in ECM degradation (Sharma and Sharma, 2007).

**Table I:** Identified Proteins by MALDI‐TOF Mass Spectrometry

Fragment number^a^	App. size (kDa)	Protein name
1	20–25	Peptidylprolyl isomerase
		FKBP3 bullous pemphigoid antigen 1 precursor
2	30	Kinesin light chain 4
		Syntrophin 5
		Immunoglobulin heavy chain variable region
3	40	Annexin A2
4	45	Fructose‐bisphosphate aldolase
		Heterogeneous nuclear ribonucleoprotein A/B
5	48–50	Beta actin
6	50–52	Translation initiation factor eIF‐4AI
7	55	Brain abundant, membrane attached signal protein 1
		Neuronal tissue‐enriched acidic protein
		Immunoglobulin heavy chain variable region
		Ubiquitin‐ specific protease USP6
8	70	Serum albumin
9	80–100	HSPA8 protein
10	120	Immunoglobulin lambda light chain variable region
		Putative homeodomain transcription factor 1 (Pdx1)
11	250	BC39498_3 zinc finger protein 682
		RET proto‐oncogene tyrosine kinase receptor
		T cell receptor delta chain

Listed are the proteins identified from the mass spectra when compared against the Matrix Science Database (MSDB). Note that there was sometimes more than one excised band per gel fragment (see Fig. 2B) which resulted in more than one protein identified per gel fragment.

a The gel fragment number corresponds to the numbering as shown in Figure 2B.

### ANXA2 INFLUENCES NUCLEAR PROTEIN COMPLEX FORMATION TO THE COL1A2 PROMOTER IN SV‐WI38 CELLS

**Figure 3:**
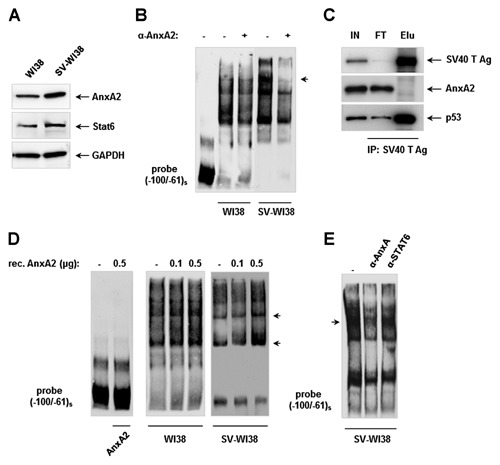
**AnxA2 is involved in complex formation to the COL1A2 promoter in SV‐WI38 cells**. (A) Western Blot showing the expression of AnxA2 and STAT6 in WI38 and SV‐WI38 cells on nuclear protein level; GAPDH expression was used as internal control. (B) EMSA experiments showing differential binding of nuclear proteins from WI38 and SV‐WI38 to the (−100/−61)_s_ probe with the effect of an anti‐AnxA2 antibody on complex formation. Major protein complexes interacting with the antibody are indicated with an arrow. (C) Western Blot of immunoprecipitated nuclear proteins prepared from SV‐WI38 cells using a SV40 T Ag antibody. p53 served as positive control for the Co‐IP assay. IN, Input fraction; FT, Flow‐through fraction; Elu, Eluate. (D) EMSA experiments showing the effect of increasing amounts of recombinant human AnxA2 protein on complex formation in the absence or presence of nuclear extracts prepared from WI38 or SV‐WI38 cells. Major protein complexes influenced by addition of recombinant AnxA2 are indicated with an arrow. (E) EMSA analysis of the influence of anti‐AnxA2 and anti‐STAT6 antibodies, respectively, on SV‐WI38 nuclear protein binding to the (−100/−61)_s_ probe. Major protein complexes interacting with the antibody are indicated with an arrow.

**Figure 4:**
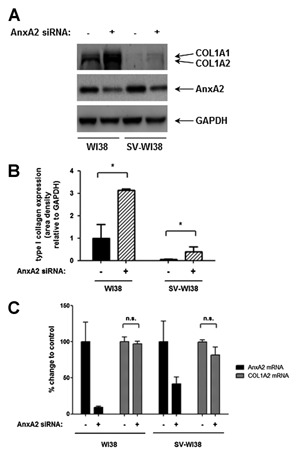
**AnxA2 regulates type I collagen expression**. (A) Representative Western Blot showing the effect of AnxA2 siRNA knock‐down on type I collagen expression in WI38 and SV‐WI38 cells. Experiments were performed in triplicates, band intensities quantified by VisionWorks® LS analysis software, normalised to GAPDH loading controls and presented relative to WI38 control which was set as 1. *P < 0.05 (B). (C) Quantitative Light Cycler RT‐PCR showing COL1A2 expression upon AnxA2 siRNA knockdown in WI38 and SV‐WI38 cells. Controls were set as 100%. Experiments were performed in triplicates and normalised to β‐actin and HSPC3 expression. n.s., not significant.

Although not primarily characterised as a transcriptional regulator, AnxA2 has been reported to have some RNA‐ and DNA‐binding properties [Boyko et al., 1994; Kwak et al., 2011; Vedeler et al., 2012] and was shown to display multiple protein interactions, among them an association and nuclear translocation of the STAT6 transcription factor [Das et al., 2010]. In order to confirm that AnxA2 is involved in complex formation to the proximal COL1A2 promoter, we performed EMSA experiments using a specific antibody against AnxA2. As shown in Figure 2C, this antibody interfered with protein complex binding to both the (3xCME)_s_ and the (−100/−61)_s_ probes resulting in diminished band intensities which confirmed that AnxA2 was part of the protein complexes bound to the proximal COL1A2 promoter. We next asked whether AnxA2 was differentially expressed upon SV40 transformation of WI38 cells and might therefore lead to differential COL1A2 transcription. Indeed, we found that AnxA2 protein levels were markedly enhanced in nuclear extracts of SV‐WI38 cells compared to untransformed WI38 cells (Fig. 3A). Interestingly, the amount of cytosolic AnxA2 was not altered in SV‐WI38 cells (Fig. 4A). Moreover, when WI38 and SV‐WI38 nuclear extracts were tested in EMSAs, we found that the AnxA2‐antibody strongly interfered with complex formation of SV‐WI38 proteins to the (−100/−61)_s_ probe while there was only a slight interaction when WI38 nuclear extracts were used (Fig. 3B). This increased accumulation of AnxA2 in SV‐WI38 cells in the context of the proximal COL1A2 promoter binding protein complex was not due to SV40 interaction as revealed by co‐immunoprecipitation experiments: while SV40 T Ag was precipitated together with p53 as expected (Simanis and Lane, 1985), AnxA2 was not found to be a SV40 T Ag binding partner (Fig. 3C).

We also observed that increasing amounts of recombinant AnxA2 to the EMSA binding reaction led to a strong increase in SV‐WI38 protein complex formation to the (−100/−61)_s_ probe while this was not observed for WI38 nuclear proteins (Fig. 3D). Interestingly, recombinant AnxA2 did not display any direct DNA binding activity (Fig. 3D), suggesting that AnxA2 might stabilise nuclear protein complexes in SV‐WI38 cells rather than bind directly to the DNA. This was indirectly supported by Chromatin Immunoprecipitation (ChIP) of formaldehyde cross‐linked chromatin using an AnxA2 antibody which did not reveal any interaction of AnxA2 with the proximal COL1A2 promoter element (Supplementary Fig. S1).

As mentioned above, AnxA2 has recently been shown to physically interact with and promote nuclear entry of STAT6 [Das et al., 2010]. Indeed, we found that nuclear localisation of STAT6 was enhanced in SV‐WI38 cells compared to WI38 cells (Fig. 3A). However, binding of STAT6 to the proximal COL1A2 promoter did not occur as determined using an antibody to STAT6 in EMSA experiments (Fig. 3E) and ChIP (Supplementary Fig. S1), therefore serving as negative control in these assays.

### ANXA2 IS INVOLVED IN THE REGULATION OF TYPE I COLLAGEN GENE EXPRESSION

In order to define a functional role for AnxA2 in type I collagen regulation, we decreased the expression of AnxA2 using siRNA. As shown in Figure 4A and B, knockdown of AnxA2 significantly increased type I collagen protein levels in both WI38 and SV‐WI38 cells, by threefold and sevenfold, respectively. However, this effect was not found to be mediated at the transcriptional level. Quantitation of COL1A2 mRNA upon AnxA2 siRNA knockdown by quantitative RT‐PCR showed that COL1A2 gene transcription was not significantly affected (Fig. 4C). The function of AnxA2 in COL1A2 regulation is therefore defined to be at the post‐transcriptional rather than at the transcriptional level.

## DISCUSSION

Previous studies of the human α2(I) collagen promoter have demonstrated that the promoter region spanning nucleotides −100 to −61 relative to the transcriptional start site is essential for transcriptional downregulation in the SV40 transformed human embryonic fibroblast cell line SV‐WI38 [Parker et al., 1989; Collins et al., 1997]. In an effort to identify the regulatory protein(s) that bind the CME in this region, we applied optimised EMSA conditions to allow binding of SV‐WI38 nuclear extracts to a single‐stranded DNA probe consisting of three repeats of the CME. The proximal regulatory COL1A2 promoter can potentially form secondary structures leaving parts of the DNA strands in the single‐stranded form [Collins et al., 1997]; indeed, we observed that binding of nuclear proteins was strongest when the EMSA‐probe spanning the regulatory COL1A2 promoter region was used in the single‐stranded form in the sense orientation. Such scenarios could be induced upon protein binding to the promoter, leaving the DNA transiently single‐stranded, and could facilitate (or abrogate) co‐factor binding to regulate gene expression. As this observation was a first indication of a larger protein complex binding to the CME instead of a single regulatory protein, we applied magnetic separation that is usually very gentle to the target proteins or protein complexes that tend to be dissociated by traditional column chromatography techniques [Safarik and Safarikova, 2004]. We identified a number of proteins with possible functions in transcriptional regulation that were bound to and eluted from the (3xCME)_s_ probe, among them AnxA2, hnRNP‐A/B, translation initiation factor eIF‐4AI, HSPA8 protein, putative homeodomain transcription factor 1 (Pdx1), and BC39498_3 zinc finger protein 682. As the 40 kDa protein band representing AnxA2 showed up as one of the dominant molecules eluted from the magnetic beads, and due to its known nuclear localisation [Das et al., 2010] and its biological function in ECM degradation [Sharma and Sharma, 2007], we considered AnxA2 as an attractive candidate involved in type I collagen regulation as part of a regulatory protein complex.

We confirmed the involvement of AnxA2 in CME binding by EMSA studies with a specific antibody against AnxA2 and observed that the antibody did not completely disrupt complex formation to the probe supporting the hypothesis of a large multi‐protein complex interacting with the CME. We further could show that AnxA2 itself did not directly bind to the single‐stranded COL1A2 promoter region (−100/−61)_s_, but that its presence led to increased protein binding to the probe. Although early work on AnxA2 suggested some DNA‐binding properties [Boyko et al., 1994], this binding was probably not directly through AnxA2 but in complex with other proteins as we observed in this study. We therefore hypothesise that AnxA2 stabilises nuclear protein complexes at the CME region as the anti‐AnxA2 antibody disrupted protein‐DNA binding to some extent while addition of recombinant AnxA2 protein increased complex formation. These effects were more pronounced with SV‐WI38 nuclear proteins compared to WI38 extracts, the latter expressing nuclear AnxA2 to a lower extent, suggesting a role of AnxA2 in the differential regulation of the COL1A2 gene in these two cell lines. Interestingly, we did not detect differences in the sequence of the AnxA2 transcript in the two cell lines (data not shown), suggesting that SV40 transformation did not cause mutations in the AnxA2 gene that could be responsible for the differential type I collagen expression [Lubbe et al., 1982]. Moreover, we ruled out SV40 T Ag interaction with AnxA2 leading to enhanced binding to the proximal COL1A2 promoter.

Functional studies confirmed the involvement of AnxA2 in type I collagen expression as altered AnxA2 expression had a substantial influence: siRNA knockdown of AnxA2 was accompanied by significantly elevated type I collagen levels in both WI38 and SV‐WI38 cells. Although this effect was more pronounced in SV‐WI38 cells, type I collagen expression could not be rescued to the level of the untransformed WI38 cells. Conversely, transient overexpression of AnxA2 slightly decreased type I collagen expression in WI38 cells which did not reach the low amounts seen in SV40‐transformed cells (data not shown), suggesting additional repressive mechanisms upon malignant transformation. Surprisingly, alterations of the amount of available AnxA2 did not affect transcription of the COL1A2 gene, suggesting that AnxA2 primarily acts via post‐transcriptional regulation of the gene. Indeed, it has been observed previously that AnxA2 can bind to cis‐acting elements in the 3′‐UTR of specific genes [Vedeler et al., 2012]. For example, a study on the post‐ transcriptional regulation of the collagen prolyl 4‐hydroxylase‐α(I) gene, that plays a central role in collagen synthesis [Kivirikko and Pihlajaniemi, 1998], identified protein complexes similar to our study that interacted with the 5′‐ and 3′‐UTRs of the gene consisting of nucleolin, ribosomal protein L7a, and eukaryotic translation elongation factor α1 (to 5′‐UTR) as well as nucleolin, splicing factor proline/glutamine‐rich, HSP8 isoform 2, members of the hnRNP family hnRNP‐R, hnRNP‐A2/B1, and hnRNP‐A3, Anx A2, and BRD3 protein (to 3'UTR), respectively. All of these proteins are known RNA‐binding proteins with AnxA2 additionally assembling with nucleolin via protein–protein interactions [Fahling et al., 2006]. Although known to be involved in RNA binding, some of the proteins identified in our study might constitute a complex that can also recognise single‐stranded DNA as described for hnRNP [Michelotti et al., 1995; Thakur et al., 2003]. It is possible that the proteins constituting the proximal COL1A2 promoter complex serve as a reservoir of post‐transcriptional regulators that are localised in close proximity to the transcriptional machinery and the emerging mRNA. The observation that AnxA2 is involved in the post‐transcriptional regulation of both the collagen prolyl 4‐hydroxylase‐α(I) and the COL1A2 gene may represent a link between different collagen synthesis pathways and the AnxA2 regulatory complex.

Although we demonstrated a novel role in the negative regulation of type I collagen gene expression, the presence of AnxA2 alone does not explain the observed substantial overall decrease of COL1A2 mRNA and protein upon SV40 transformation. AnxA2 acting at the post‐transcriptional level contributes to type I collagen down‐regulation; however, transcriptional mechanisms do certainly play a significant role in the overall negative regulation. The question of the identity of the proteins that bind to the CME resulting in inhibition of COL1A2 transcription in SV‐WI38 cells still remains. A possible candidate that has been shown to directly interact with AnxA2 is the transcription factor STAT6, leading to enhanced transcriptional activity [Das et al., 2010]. Interestingly, the observed AnxA2‐promoted nuclear entry of STAT6 upon IL4 treatment [Das et al., 2010] can also be linked to COL1A2 regulation. A previous study demonstrated that STAT6 activated by IL‐4 can bind to various parts of the COL1A2 promoter and contributes to the combined action of SP1 and NFκB (all of which are mediated by IL‐4) to activate COL1A2 transcription [Buttner et al., 2004]. However, the potential STAT6 binding sites identified in that study [Buttner et al., 2004] were not located in the −100/−61 COL1A2 promoter element. Although we found that STAT6 was increased in SV‐WI38 nuclei, we could not confirm its involvement as AnxA2 binding partner (not shown) or as part of the protein complex binding to the promoter region −100/−61.

The role of AnxA2 in the carcinogenesis of human cancers is still debated as it has been shown to be overexpressed in some cancers and downregulated in others, both of which have been identified as beneficial to cancer cells depending on location (Liu et al., 2003; Zhai et al., 2011). AnxA2 has been shown to be involved in ECM degradation and ECM remodelling through the activation of serine proteases. This activity is vital during wound repair processes, when cells need to differentiate, proliferate and migrate in order to regenerate tissue, but can be co‐opted by cancer cells to increase their invasive potential, thereby playing a direct role in the malignant phenotype of tumour cells [Sharma and Sharma, 2007; Hitchcock et al., 2014]. The role of AnxA2 in the regulation of the COL1A2 gene in SV40‐transformed cells as demonstrated in this study can be partly ascribed to its nuclear accumulation where it is assumed to play a role in 3′UTR‐mRNA binding leading to post‐transcriptional repression. Although constituting a protein complex bound to the minimal −100/−61 region of the human COL1A2 promoter, AnxA2 was not found to exert its effect on transcription. It is also possible that AnxA2 plays a role in the nuclear export of the COL1A2 transcript as has been shown for c‐myc, collagen prolyl 4‐hydroxylase‐α (I) and its own cognate mRNA transcript [Mickleburgh et al., 2005; Fahling et al., 2006; Hollas et al., 2006]. Together with reduced mRNA expression of COL1A2 upon SV40 transformation, multi‐protein complex associated‐AnxA2 may interact with the COL1A2 mRNA transcript and prevent nuclear export and translation of the mRNA transcript.

## CONCLUSION

AnxA2 is known to be associated with diverse cellular processes including functions in ECM degradation through intercellular and cell‐ECM interactions. Here, we describe a novel function for nuclear AnxA2 as part of a post‐transcriptional regulatory protein complex involved in the regulation of type I collagen. Its regulatory function was more pronounced upon transformation with the SV40 tumour virus which led to nuclear AnxA2 accumulation. We speculate that, under normal conditions, AnxA2 is involved in physiological COL1A2 regulation to prevent excessive deposition of type I collagen which is characteristic of many fibrotic disorders such as systemic sclerosis, hepatic cirrhosis and pulmonary fibrosis [Varga and Jimenez, 1995]. However, upon malignant transformation which is characterised by ECM degradation and down‐regulation of ECM components, the AnxA2‐mediated repressive functions in the context of type I collagen expression are enhanced, contributing to decreased type I collagen synthesis in favour of cancer development and progression.

## Supporting information

Supporting Information.

## References

[jcb24989-bib-0001] Boyko V , Mudrak O , Svetlova M , Negishi Y , Ariga H , Tomilin N . 1994 A major cellular substrate for protein kinases, annexin II, is a DNA‐binding protein. FEBS Lett 345:139–142. 8200445 10.1016/0014-5793(94)00419-6

[jcb24989-bib-0002] Buttner C , Skupin A , Rieber EP . 2004 Transcriptional activation of the type I collagen genes COL1A1 and COL1A2 in fibroblasts by interleukin‐4: Analysis of the functional collagen promoter sequences. J Cell Physiol 198:248–258. 14603527 10.1002/jcp.10395

[jcb24989-bib-0003] Collins M , Leaner VD , Madikizela M , Parker MI . 1997 Regulation of the human alpha 2(1) procollagen gene by sequences adjacent to the CCAAT box. Biochem J 322((Pt 1)):199–206. 9078262 10.1042/bj3220199 PMC1218177

[jcb24989-bib-0004] Collins M , Smith AA , Parker MI . 1998 Characterization of two distinct families of transcription factors that bind to the CCAAT box region of the human COL1A2 gene. J Cell Biochem 70:455–467. 9712144

[jcb24989-bib-0005] Das S , Shetty P , Valapala M , Dasgupta S , Gryczynski Z , Vishwanatha JK . 2010 Signal transducer and activator of transcription 6 (STAT6) is a novel interactor of annexin A2 in prostate cancer cells. Biochemistry 49:2216–2226. 20121258 10.1021/bi9013038

[jcb24989-bib-0006] de Haan JB , Gevers W , Parker MI . 1986 Effects of sodium butyrate on the synthesis and methylation of DNA in normal cells and their transformed counterparts. Cancer Res 46:713–716. 2416432

[jcb24989-bib-0007] Dickson LA , de Wet W , Di Liberto M , Weil D , Ramirez F . 1985 Analysis of the promoter region and the N‐propeptide domain of the human pro alpha 2(I) collagen gene. Nucleic Acids Res 13:3427–3438. 4011429 10.1093/nar/13.10.3427 PMC341250

[jcb24989-bib-0008] Fahling M , Mrowka R , Steege A , Nebrich G , Perlewitz A , Persson PB , Thiele BJ . 2006 Translational control of collagen prolyl 4‐hydroxylase‐alpha(I) gene expression underhypoxia. J Biol Chem 281:26089–26101. 16837461 10.1074/jbc.M604939200

[jcb24989-bib-0009] Fenhalls G , Geyp M , Dent DM , Parker MI . 1999 Breast tumour cell‐induced down‐regulation of type I collagen mRNA in fibroblasts. Br J Cancer 81:1142–1149. 10584874 10.1038/sj.bjc.6690821 PMC2374322

[jcb24989-bib-0010] Hitchcock JK , Katz AA , Schäfer G . 2014 Dynamic reciprocity: the role of annexin A2 in tissue integrity. J Cell Commun Signal 8:125–133. 24838661 10.1007/s12079-014-0231-0 PMC4063989

[jcb24989-bib-0011] Hollas H . 2006 Annexin A2 recognises a specific region in the 3'‐UTR of its cognate messenger RNA. Biochim Biophys Acta 1763:1325–1334. 17045350 10.1016/j.bbamcr.2006.08.043

[jcb24989-bib-0012] Jinka R . 2012 Alterations in cell‐extracellular matrix interactions during progression of cancers. Int J Cell Biol 2012:219196. 22262973 10.1155/2012/219196 PMC3259478

[jcb24989-bib-0013] Kivirikko KI , Pihlajaniemi T . 1998 Collagen hydroxylases and the protein disulfide isomerase subunit of prolyl 4‐hydroxylases. Adv Enzymol Relat Areas Mol Biol 72:325–398. 9559057 10.1002/9780470123188.ch9

[jcb24989-bib-0014] Krainova NA , Khaustova NA , Makeeva DS , Fedotov NN , Gudim EA , Ryabenko EA , Shkurnikov MU , Galatenko VV , Sakharov DA , D.V M . 2013 Evaluation of potential reference genes for qRT–PCR data normalization in hela cells. Appl Biochem Microbiol 49:743–749.

[jcb24989-bib-0015] Kwak H , Park MW , Jeong S . 2011 Annexin A2 binds RNA and reduces the frameshifting efficiency of infectious bronchitis virus. PloS ONE 6:e 24067. 10.1371/journal.pone.0024067 PMC3168876 21918681

[jcb24989-bib-0016] Leaner VD , Masemola A , Parker MI . 2005 Species‐specific regulation of the alpha‐2(I) procollagen gene by proximal promoter elements. IUBMB Life 57:363–370. 16036621 10.1080/15216540500092039

[jcb24989-bib-0017] Liu J , Rothermund CA , Ayala‐Sanmartin J , Vishwanatha JK . 2003 Nuclear annexin II negatively regulates growth of LNCaP cells and substitution of ser 11 and 25 to glu prevents nucleo‐cytoplasmic shuttling of annexin II. BMC biochemistry 4:10. 12962548 10.1186/1471-2091-4-10 PMC200965

[jcb24989-bib-0018] Lubbe L , Strauss M , Scherneck S , Geissler E . 1982 The DNA tumor virus SV 40 induces gene mutations in human cells. Reversion of HPRT deficiency. Human Genet 61:236–241. 6293958 10.1007/BF00296449

[jcb24989-bib-0019] Meerbach A , Gruhn B , Egerer R , Reischl U , Zintl F , Wutzler P . 2001 Semiquantitative PCR analysis of Epstein‐Barr virus DNA in clinical samples of patients with EBV‐associated diseases. J Med Virol 65:348–357. 11536243 10.1002/jmv.2040

[jcb24989-bib-0020] Michelotti EF , Tomonaga T , Krutzsch H , Levens D . 1995 Cellular nucleic acid binding protein regulates the CT element of the human c‐myc protooncogene. J Biol Chem 270:9494–9499. 7721877 10.1074/jbc.270.16.9494

[jcb24989-bib-0021] Mickleburgh I , Burtle B , Hollas H , Campbell G , Chrzanowska‐Lightowlers Z , Vedeler A , Hesketh J . 2005 Annexin A2 binds to the localization signal in the 3' untranslated region of c‐ myc mRNA. FEBS J 272:413–421. 15654879 10.1111/j.1742-4658.2004.04481.x

[jcb24989-bib-0022] Parker MI , Smith AA , Gevers W . 1989 Absence of alpha 2(1) procollagen synthesis in a clone of SV40‐transformed WI‐38 human fibroblasts. J Biol Chem 264:7147–7152. 2540177

[jcb24989-bib-0023] Parker MI , Smith AA , Mundell K , Collins M , Boast S , Ramirez F . 1992 The abolition of collagen gene expression in SV40‐transformed fibroblasts is associated with trans‐acting factor switching. Nucleic Acids Res 20:5825–5830. 1333588 10.1093/nar/20.21.5825 PMC334422

[jcb24989-bib-0024] Rossert J , Terraz C , Dupont S . 2000 Regulation of type I collagen genes expression. Nephrol Dial Transplant 15(Suppl 6):68. 10.1093/ndt/15.suppl_6.66 11143996

[jcb24989-bib-0025] Safarik I , Safarikova M . 2004 Magnetic techniques for the isolation and purification of proteins and peptides. Biomagn Res Technol 2:7. 15566570 10.1186/1477-044X-2-7 PMC544596

[jcb24989-bib-0026] Sharma MC , Sharma M . 2007 The role of annexin II in angiogenesis and tumor progression: A potential therapeutic target. Curr Pharm Des 13:3568–3575. 18220793 10.2174/138161207782794167

[jcb24989-bib-0027] Simanis V , Lane DP . 1985 An immunoaffinity purification procedure for SV40 large T antigen. Virology 144:88–100. 2998049 10.1016/0042-6822(85)90308-3

[jcb24989-bib-0028] Tabata A , Namba F , Yamada M , Hasegawa T , Nakahira K , Hamada D , Kitajima H , Fukusaki E , Yanagihara I . 2006 Expression and purification of recombinant human annexin A2 in Pichiapastoris and utility of expression product for detecting annexin A2 antibody. J Biosci Bioeng 101:190–197. 16569618 10.1263/jbb.101.190

[jcb24989-bib-0029] Thakur S , Nakamura T , Calin G , Russo A , Tamburrino JF , Shimizu M , Baldassarre G , Battista S , Fusco A , Wassell RP , Dubois G , Alder H , Croce CM . 2003 Regulation of BRCA1 transcription by specific single‐stranded DNA binding factors. Mol Cell Biol 23:3774–3787. 12748281 10.1128/MCB.23.11.3774-3787.2003 PMC155225

[jcb24989-bib-0030] van Rooyen BA , Schäfer G , Leaner VD , Parker MI . 2013 Tumour cells down‐regulate CCN2 gene expression in co‐cultured fibroblasts in a Smad7‐ and ERK‐dependent manner. Cell communication and signaling: CCS 11:75. 24090133 10.1186/1478-811X-11-75 PMC3850759

[jcb24989-bib-0031] Varga J , Jimenez SA . 1995 Modulation of collagen gene expression: Its relation to fibrosis in systemic sclerosis and other disorders. Ann Intern Med 122:60–62. 7985897 10.7326/0003-4819-122-1-199501010-00010

[jcb24989-bib-0032] Vedeler A , Hollas H , Grindheim AK , Raddum AM . 2012 Multiple roles of annexin A2 in post‐ transcriptional regulation of gene expression. Curr Protein Pept Sci 13:401–412. 22708494 10.2174/138920312801619402

[jcb24989-bib-0033] Zhai H , Acharya S , Gravanis I , Mehmood S , Seidman RJ , Shroyer KR , Hajjar KA , Tsirka SE . 2011 Annexin A2 promotes glioma cell invasion and tumor progression. J Neurosci 31:14346–14360. 21976520 10.1523/JNEUROSCI.3299-11.2011 PMC3201988

