# Supplementary material for: A Novel Role of Annexin A2 in Human Type I Collagen Gene Expression
Source: J Cell Biochem. 2015 Jan 20;116(3):408–17. doi: 10.1002/jcb.24989 (PMC4988497; doi:10.1002/jcb.24989)
Supplement: Supplementary file 1 — Supporting Information. [file JCB-116-408-s001.docx]

**Supplementary Materials and Methods**

***Chromatin Immunoprecipitation (ChIP)***

SV‐WI38 cells were grown to approximately 80% confluency and chromatin prepared for ChIP analysis as described in detail before (van der Watt et al., 2011), where chromatin fragments with an average size of 250‐1500bp were recovered after sonication (Fig. S1A). Chromatin extracts, pre‐ cleared with Protein G Plus Agarose beads (Santa Cruz), were precipitated with 2µg of either anti‐ Annexin A2 (BD Biosciences) or anti‐Stat6 (BD Biosciences), both being described suitable for ChIP application (Das et al., 2010). Immunoprecipitation of chromatin fragments prepared from WI38 cells using the antibody anti‐Sp1 (H‐225) (Santa Cruz) served as positive control. Immunocomplexes were collected, washed, eluted and reverse cross‐linked as described (van der Watt et al., 2011), thereafter DNA was purified using the QIAamp DNA Mini kit (Qiagen) and analysed by PCR using 1µl of either the precipitated DNA or the corresponding input sample. The PCR primers (F: *5’ CAAATTCTGCCCATGTCGGG 3’*; R: *5’ AGACTCCTTGTGTCGCAGAG 3’*) were designed to span the

proximal COL1A2 promoter region from ‐372 to +118, comprising the CME region as well as several Sp1 sites upon which basal COL1A2 promoter activity is highly dependent (Dzobo et al., 2012).

Das, S., P. Shetty, M. Valapala, S. Dasgupta, Z. Gryczynski, and J.K. Vishwanatha. 2010. Signal transducer and activator of transcription 6 (STAT6) is a novel interactor of annexin A2 in prostate cancer cells. *Biochemistry*. 49:2216‐2226.

Dzobo, K., V.D. Leaner, and M.I. Parker. 2012. Feedback regulation of the alpha2(1) collagen gene via the Mek‐Erk signaling pathway. *IUBMB life*. 64:87‐98.

van der Watt, P.J., E. Ngarande, and V.D. Leaner. 2011. Overexpression of Kpnbeta1 and Kpnalpha2 importin proteins in cancer derives from deregulated E2F activity. *PloS one*. 6:e27723.


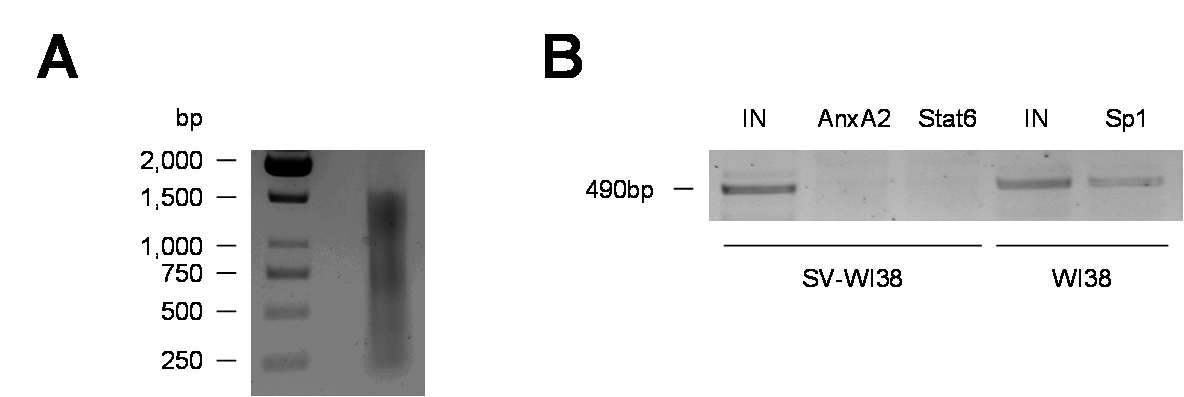


**Figure S1. Annexin A2 does not bind to the proximal COL1A2 promoter region.**

(A) Agarose gel electrophoresis of sheared chromatin prepared from SV‐WI38 cells indicating DNA fragment sizes between 250bp and 1500bp. (B) ChIP of chromatin prepared from SV‐WI38 cells using an anti‐AnxA2 antibody or Stat6 antibody (negative control) as indicated; ChIP of WI38 chromatin using an anti‐Sp1 antibody served as positive control. The recovered DNA and the corresponding control input DNA were amplified by PCR using primers spanning the proximal COL1A2 promoter region from ‐372 to +118, and the PCR products were resolved by agarose gel electrophoresis.
